# The costs and cost effectiveness of providing second-trimester medical and surgical safe abortion services in Western Cape Province, South Africa

**DOI:** 10.1371/journal.pone.0197485

**Published:** 2018-06-28

**Authors:** Naomi Lince-Deroche, Deborah Constant, Jane Harries, Judith Kluge, Kelly Blanchard, Edina Sinanovic, Daniel Grossman

**Affiliations:** 1 Ibis Reproductive Health, Johannesburg, South Africa; 2 Women’s Health Research Unit, School of Public Health and Family Medicine, University of Cape Town, Cape Town, South Africa; 3 Department of Obstetrics & Gynaecology, University of Stellenbosch and Tygerberg Hospital, Cape Town, South Africa; 4 Ibis Reproductive Health, Cambridge, Massachusetts, United States of America; 5 Health Economics Unit, School of Public Health and Family Medicine, University of Cape Town, Cape Town, South Africa; Public Library of Science, UNITED KINGDOM

## Abstract

**Background:**

In South Africa, access to second-trimester abortion services, which are generally performed using medical induction with misoprostol alone, is challenging for many women. We aimed to estimate the costs and cost effectiveness of providing three safe second-trimester abortion services (dilation and evacuation (D&E)), medical induction with mifepristone and misoprostol (MI-combined), or medical induction with misoprostol alone (MI-misoprostol)) in Western Cape Province, South Africa to aid policymakers with planning for service provision in South Africa and similar settings.

**Methods:**

We derived clinical outcomes data for this economic evaluation from two previously conducted clinical studies. In 2013–2014, we collected cost data from three public hospitals where the studies took place. We collected cost data from the health service perspective through micro-costing activities, including discussions with site staff. We used decision tree analysis to estimate average costs per patient interaction (e.g. first visit, procedure visit, etc.), the total average cost per procedure, and cost-effectiveness in terms of the cost per complete abortion. We discounted equipment costs at 3%, and present the results in 2015 US dollars.

**Results:**

D&E services were the least costly and the most cost-effective at $91.17 per complete abortion. MI-combined was also less costly and more cost-effective (at $298.03 per complete abortion) than MI-misoprostol (at $375.31 per complete abortion), in part due to a shortened inpatient stay. However, an overlap in the plausible cost ranges for the two medical procedures suggests that the two may have equivalent costs in some circumstances.

**Conclusion:**

D&E was most cost-effective in this analysis. However, due to resistance from health care providers and other barriers, these services are not widely available and scale-up is challenging. Given South Africa’s reliance on medical induction, switching to the combined regimen could result in greater access to second-trimester services due to shorter inpatient stays without increasing costs.

## Introduction

Abortion is legal in South Africa on demand through 12 weeks of gestation and for reasons including socioeconomic grounds through 20 weeks of gestation [1]. Despite the country’s liberal law, however, there are still many barriers to accessing abortion services, especially second-trimester services [2–5]. Yet many women present late or experience systemic barriers and ultimately obtain their abortion in the second trimester [6,7]. More than 20% of South Africa’s total annual abortions in the public sector are performed between 13 and 20 weeks gestation [5,8]. This proportion is high when compared to England and the United States where less than ten percent of abortions are performed in the second trimester [9,10].

Today South Africa’s Standard Treatment Guidelines recommend inpatient medical induction for second-trimester abortion [11]. Doctors perform the initial assessment and write a prescription for the required medication. Then nurses often manage the induction process until expulsion when the doctor will return to verify completion. If evacuation is required for retained products, that is also performed by a doctor. Mifepristone was added to the national medical induction guidelines in 2012 [12]; however many public facilities have been slow to convert their practice, and misoprostol-only medical induction is still the only available option in many settings. Concerns about the high cost of mifepristone [13,14] may have contributed to this slow transition.

Dilation and evacuation (D&E) services are also offered in South Africa, albeit on a very limited basis, including at a few public facilities in Western Cape Province and some private facilities such as clinics run by Marie Stopes South Africa. In 2011, a cross-sectional study comparing public-sector medical induction and D&E services in Western Cape Province by Grossman et al. showed that both procedures were performed safely and that women were generally satisfied with their experience [7]. D&E services required much less time than medical induction services, and as a result, service volume was higher at D&E facilities [7].

A more recent cross-sectional study compared medical induction services with and without mifepristone–also at public facilities in Western Cape Province [15]. The authors found that the addition of mifepristone to the medical induction regimen shortened procedure times and reduced hospitalization, increasing the capacity of the facilities to provide services [15]. Shortening procedure times at facilities across the nation could help to improve access to services. More efficient spending of limited resources in the public sector, which provides abortion services free of charge to South African citizens [16], could also improve access.

In this study, we aimed to estimate the costs and cost effectiveness of providing three second-trimester abortion services: medical induction with and without mifepristone and D&E services, in the public sector in Western Cape Province. Ultimately, we aim to assist policy makers in South Africa and similar settings with decision-making and contribute to improved abortion access.

## Materials and methods

### Clinical service provision and statistics

**Fig 1:**
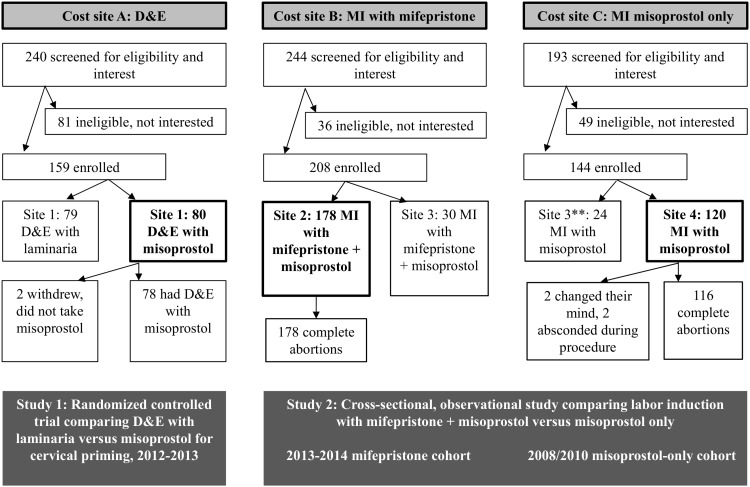
Three facilities in Western Cape Province, South Africa providing data for this cost evaluation. * D&E = dilation and evacuation, MI = medical induction, “with mifepristone” = mifepristone plus misoprostol *Bold boxes indicate data/sites included in this cost evaluation. ** This site 3 is the same site 3 that switched to a mifepristone plus misoprostol regimen in the 2013–2014 mifepristone plus misoprostol cohort.

Wherever possible we derived data on clinical service provision and outcomes for this analysis from two previously conducted clinical studies. The first study was a randomized controlled trial comparing D&E performed with misoprostol versus laminaria for cervical priming; it was conducted at one public hospital (Fig 1, site 1) in Western Cape Province in 2012–2013 [17]. For this analysis, we used the service parameters and outcomes as documented for the misoprostol D&E arm of the trial. The second study, also conducted in Western Cape Province, utilized repeated, cross-sectional observations over time to compare services offering medical induction with misoprostol only versus a combined regimen of misoprostol plus mifepristone [15]. For this second study, data were collected at three public hospitals, though only two are included in this cost evaluation. One hospital (Fig 1, site 2) offered the combined regimen throughout 2013–2014. An additional hospital (Fig 1, site 4) offered medical induction with misoprostol only in 2008 and 2010. Site 3 was excluded for logistical reasons.

In the D&E clinical trial (Fig 1, study 1) [17], all women had an initial visit at the hospital where they requested the abortion service and went through a standard evaluation with a doctor to determine eligibility for a second-trimester abortion. If eligible for the study, a facility nurse provided an appointment for the D&E procedure on a subsequent day (which is the local standard of care). Before leaving the facility, the nurse gave the women misoprostol and instructed them to administer the medication buccally at home early in the morning on their procedure day. A doctor performed the D&E study procedures during the facility’s routine D&E service, one after the other on a specific weekday. Following the procedure, the women waited in a recovery area where a nurse checked their vital signs before they were allowed to leave the facility. For the D&E study participants, a study staff member attempted to conduct a follow-up interview one week after the procedure. However, follow-up post D&E is not the local standard of care.

In the observational study (Fig 1, study 2), all medical induction services required at least two visits to the study facilities: the initial visit where a consultation and exam were performed and an inpatient visit for the medical induction procedure [15]. Women who had medical induction with the combined regimen were instructed to take the mifepristone at home 24–48 hours before admission for their inpatient visit. Then, during the inpatient visit, facility nurses gave all women misoprostol in 3 to 4 hourly doses until fetal expulsion. In the misoprostol-only group, the initial dose was generally 600 μg followed by doses of 200 μg or 400 μg. If expulsion had not occurred after 13 total doses, the woman was allowed to rest, and then the misoprostol was started again. If that approach was still unsuccessful, other prostaglandins were used. For the combined mifepristone-misoprostol regimen, the first misoprostol dose varied from 600 to 800 μg, and later doses (up to 10 if needed) were generally 400 μg [15]. After expulsion, verification of completion was done by a doctor (who sometimes performed an additional evacuation), and women were discharged from the hospital. No follow-up visits were required or conducted.

For both the clinical trial and the observational study, clinical and procedural outcome data were collected by trained study staff through prospective medical file reviews and structured patient interviews before and immediately after the abortion procedure. The possible procedural outcomes were: 1) complete abortion without need for additional evacuation, 2) complete abortion achieved via the initial procedure plus a subsequent evacuation, and 3) an incomplete procedure at study exit (due to the woman leaving or being transferred to another facility prior to procedure completion). The two complete abortion categories were further broken down into uncomplicated or complicated procedures, which included hemorrhage, sepsis, shock, laceration or perforation. “Extra” evacuations were also defined as complications [18]. For the purposes of this analysis, we also defined any D&E women who experienced fetal expulsion prior to the planned D&E procedure but who had an evacuation anyway using the same aspirator, cannulas, etc. used for the D&E service as having had an “extra” evacuation. Medical induction women who had an evacuation after fetal expulsion, again using an aspirator and cannulas, were also defined as having had an “extra” evacuation.

Ethics approvals for all studies–including both clinical and cost data collection–were obtained from Allendale Investigational Review Board and the Human Research Ethics Committee at the University of Cape Town. The clinical trial protocol was also approved by the Human Research Ethics Committee at Stellenbosch University and the South African Medicines Control Council. The clinical trial was registered at ClinicalTrials.gov (registration number NCT01597726). All women who participated in both studies provided written informed consent.

### Costing

We conducted micro-costing from the health service perspective at two hospitals—the one offering D&E services (Fig 1, site 1) and one offering medical induction services with mifepristone and misoprostol (site 2). Micro-costing, sometimes referred to as “bottom-up” costing, generally involves determining all resources used when offering a particular intervention and then multiplying the resource usage, or volume, by the cost per resource type to obtain a total cost. For this analysis, we included staff types and average time requirements, equipment, consumables, medication, and laboratory tests.

We visited study sites 1 and 2 in 2013–2014 and, through discussions with staff who regularly worked in the abortion services, we collected data on the resources required to perform the abortions. At the D&E site, the randomized controlled trial had finished enrollment when we visited for costing; however, the nurses and doctors at the site continued to provide the D&E service in the same way as during the trial. Unfortunately, due to changes in service provision at the third study site (Fig 1, site 4), which ultimately began outsourcing services, we are not able to conduct a full micro-costing exercise at the facility. However, we were able to verify differences and similarities between the mifepristone-plus-misoprostol service at site 2 and the misoprostol-only service at site 4 based on detailed descriptive service data collected at the misoprostol-only service in 2008 [7] and discussions with staff at site 4 in 2013.

After collecting resource usage data for all three sites, we then used facility expenditure records, information from medical suppliers, and publicly available sources to determine the unit costs for the resources used [19–21]. We annualized capital costs based on depreciation periods recommended by the South Africa Revenue Service [22] at a discount rate of 3%. Costs were collected in South African Rand, inflated to 2015 prices using the Consumer Price Index [23], and are reported here in 2015 US dollars based on an average exchange rate for 2015 of 14.39 Rands per dollar [24].

The D&E service is an outpatient service. We assumed that all required resources were divisible as needed. For example, if 15 minutes of a gynecologist’s time were needed, then only 15 minutes were included in the abortion procedure cost, and it was assumed that the rest of the gynecologist’s time was covered by other duties or services. We assumed that equipment—e.g. beds, desks, and other furniture and machines—were used exclusively for abortion services. We based this assumption on the tendency to segregate abortion services from other obstetric and gynecological services in South African facilities. Then we estimated a monthly cost per item (after annualization and inflation of the purchase price) and divided the monthly cost by the median number of procedures performed each month in order to obtain the average cost per procedure. We used the same approach for the medical induction services with one exception. Medical induction services require inpatient admission to a hospital and nursing supervision throughout the procedure. Multiple patients are managed at once as the inpatient areas designated for abortion services have multiple beds. Thus to estimate the cost for nursing staff for medical induction, we multiplied the hourly cost for nursing staff by the median inpatient time per medical induction service type and divided that by the number of beds in the abortion areas (assuming all were in use and the nursing team shared their time across the patients).

We have excluded training costs because at the time of the study training on provision of second-trimester abortion services in South Africa was provided by an NGO as a short course after completion of one’s nursing or medical education. We have also excluded the costs of providing post-abortion contraception (which is budgeted for separately), and women’s costs for accessing second-trimester services (which have been reported elsewhere [25]). Overhead and infrastructural costs were not collected during the micro-costing exercise due to expected wide variability across facilities and to allow for comparability of the estimated average procedural costs across facilities. This means the costs of the spaces in which the procedures were performed, utilities and some personnel (e.g. receptionists, security guards, etc.) were excluded.

Complications were rare in the studies, and our costing schedule did not allow for collection of complication-related cost data in real time. Instead, we estimated complication costs as follows. For “extra” evacuations as defined above for D&E and medical induction women, we have assumed that the evacuation cost was the same as the cost for a single uncomplicated D&E. For other complications (e.g. perforation, hemorrhage, infection, etc.), we have used hospital charges published in South Africa’s Uniform Patient Fee Schedule (UPFS) [26]. For all complications except uterine perforation, we used the published costs for 24-hour hospitalization and staffing. For perforation and the required corrective surgery, which is specifically listed in the UPFS, we assume a 48-hour hospital stay, anesthesia, theater costs, and staffing. All UPFS charges reportedly include a “hotel” or facility service fee and other equipment, supplies, medications and blood products as needed [27].

### Analysis

We used Stata (Release 14. College Station, TX: StataCorp LP) to analyze the datasets from the two clinical studies. From these, we established service volume during the studies, pregnancy testing and ultrasound usage rates, medications provided, procedure duration, admission duration for medical induction, procedure outcomes, and complication rates (which were used to determine the hospitalization rate for complications).

**Fig 2:**
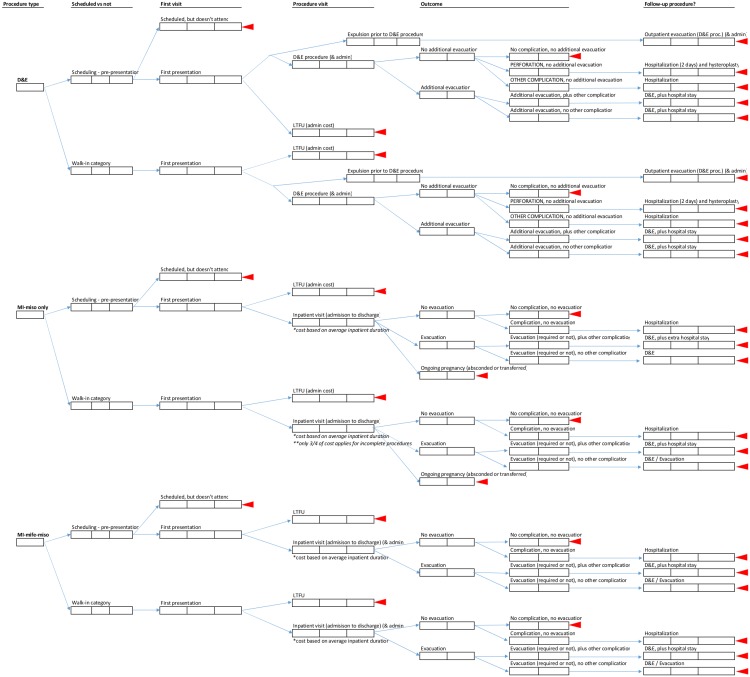
Decision tree for analysis of D&E and medical induction, based on two prior clinical studies [15,17]. * D&E = Dilation and evacuation, MI-miso = Medical induction with misoprostol only, MI-mife = Medical induction with mifepristone and misoprostol, LTFU = Lost-to-follow-up.

We then captured all required clinical and cost data in a model built for this analysis in Microsoft Excel (Microsoft Corporation 2013). The model contains: 1) source data and unit costs, 2) resource usage data collected through the micro-costing activities and analysis of the study databases, and 3) worksheets showing analytical work and outcomes. Within the model, we constructed a decision tree to facilitate visualization of all possible outcomes (Fig 2) and estimation of the total average cost per procedure. A full listing of the model contents is available in S1 Workbook snapshot.

We present the total average cost per women seen in each procedure group given the complication and completion rates as reported in the clinical studies. Costs are provided with a breakdown by cost type (i.e. personnel, medications, laboratory tests, consumables, equipment, and UPFS personnel and facilities fees). We also show the average costs for complicated and uncomplicated procedures and provide an indication of their contribution to the overall total average costs. For cost effectiveness, we defined the outcome as the cost per complete abortion, whether achieved through the initial procedure alone or the initial procedure plus an extra evacuation.

To address uncertainty in the model inputs, we estimate ranges around the base case cost estimates. The base case cost estimates represent documented resource usage multiplied by resource costs before variation for uncertainty analysis. The “uncertainty ranges” then represent costs when personnel time and supply, equipment, and hospitalization costs are adjusted by ± 25%. We did not vary costs for staff (i.e. monthly salaries), medication and laboratory tests because these costs are published publicly in South Africa on an annual basis.

Finally, we conducted univariate and bivariate sensitivity analysis to assess the relative contribution and importance of various model inputs to the cost and cost-effectiveness outcomes.

## Results

### Service parameters and resource utilization

Table 1 provides clinical and service parameters for the three procedure types (D&E, medical induction with mifepristone plus misoprostol, and medical induction with misoprostol alone). Women who had a D&E procedure all received pain medication—during and after their procedure. Women undergoing medical induction were less likely to receive pain medication. Provision of prophylactic antibiotics was common at all sites.

**Table 1:** Clinical and service parameters for second-trimester safe abortion services at three public facilities in Western Cape, South Africa.

	Site A: D&E with misoprostol for priming (2012–2013)	Site B: Medical induction with mifepristone + misoprostol (2013–2014)	Site C: Medical induction with misoprostol only (2008 / 2010)
Total women enrolled (n)	80^a^	178	120
Study duration (months)	14	9	9
Abortions monthly (median [IQR])	11 [6–15]^a^	19 [11–26]	13 [7–18]
Intake testing:			
Pregnancy test	99%	100%^b^	100%^b^
Ultrasound for dating	97%	100%	100%
Blood pressure/temperature	100%^b^	100%^b^	100%^c^
Hemoglobin	100%^b^	100%^b^	100%^c^
Full blood count, incl. blood type	0%^b^	30%^b^	30%^c^
Syphilis	0%^b^	100%^b^	100%^c^
Rhesus	90%^c^	100%^b^^,^^d^	100%^c^^,^^d^
Medications received:			
Mifepristone (1 x 200 mg PO)	N/A	100%	N/A
Misoprostol doses^e^			
1	49%	7%	1%
2	51%	21%	5%
3–5	--	58%	69%
6–10	--	12%	14%
>10	--	2%	11%
Total miso. μg’s (median [IQR])	800 [400–800]	1600 [1200–2000]	1800 [1400–2200]
Paracervical block	100%	N/A	N/A
Other analgesia	100%	83%	44%
Anti-emetic	0%	17%	3%
Other uterotonic medication	9%	99%	19%
Antibiotics	100%	90%	89%
Vitamins/minerals^f^	0%	33%	15%
Anti-immunoglobulin injection	1%	2%	1%
Gestational age at procedure (median [IQR]) (weeks)	14.7 [13.7–15.6]	17.6 [16.6–18.4]	13.6 [12.0–15.1]
Procedure duration (median [IQR])^g^	9 [8–11] minutes	11 [9–16] hours	25 [18–37] hours
Admission length (median [IQR])	N/A	28 [26–30] hours	45 [26–52] hours
Complete abortion at study exit^h^	97%	100%	97%
Procedure details/complications:			
Expulsion prior to D&E	3%	N/A	N/A
Additional evacuation performed^i^	0%	75%	57%
Uterine perf. or cervical tear	1%	1%	0%
Infection	0%	1%	0%
Hemorrhage, without transfusion	0%	0%	1%
Hemorrhage, with transfusion	0%	3%	6%
Other complication	0%	1%^j^	0%
Pre-discharge testing:			
Blood pressure/temperature	100%^b^	100%^b^	100%^c^
Hemoglobin	0%^b^	100%^b^	100%^c^
Full blood count	0%^b^	100% ^b^	100%^c^

D&E = dilation and evacuation, IQR = interquartile range, incl. = including, PO = oral, perf. = perforation

^a^ During the study 159 D&E procedures were performed (79 with laminaria and 80 with misoprostol for cervical priming). The median presented represents all procedures, laminaria and misoprostol.

^b^ Values represent reports on standard practice from the nurses or medical specialists at the sites.

^c^ We have assumed these values to be the same as those reported at site B, in part based on conversations with staff at site C.

^d^ At Site A, Rhesus testing was done using a card/testing kit. At sites B and C, blood was sent to the national laboratory service for testing.

^e^ Misoprostol doses for prior to D&E were 400 μg each. During medical induction the initial dose was usually 600 μg, and subsequent doses were 200 or 400 μg each.

^f^ Included vitamin C, folic acid, or iron supplements.

^g^ For D&E the procedure length was defined as “speculum in to speculum out.” For medical induction, procedure length was measured from the first dose of medication to fetal expulsion.

^h^ Completion was defined as leaving the study facility having had a complete abortion. This included cases with extra evacuation. The denominator is all women enrolled into the procedure group.

^i^ In many cases, the extra evacuation was performed without formal assessment of procedure completion.

^j^ One woman experienced a seizure which was determined to be unrelated to the procedure.

The D&E procedure required a median of 9 minutes [Interquartile Range (IQR) 8–11] from “speculum in” to “speculum out.” However, considering all activities related to D&E including administrative tasks, the average professional nurse spent 74 minutes per procedure (S1 Workbook, Dashboard). All women undergoing medical induction services spent more than 24 hours in the hospital during their inpatient visit; however, women undergoing medical induction with the misoprostol only regimen were admitted for considerably longer. Abortion completion was nearly 100% for all procedure types. Two women (3%) at the D&E site changed their mind and did not have the scheduled procedure, and four women (3%) at the misoprostol only site either required transfer to another facility or changed their mind about having the procedure and did not complete the dosing schedule.

Extra evacuations after medical induction were common. At both medical induction facilities, evacuation was often performed as a matter of routine, whether expulsion appeared complete or not. Expulsion prior to D&E and other complications for both D&E and medical induction were rare events.

The resources required per procedure are outlined in Table 2. Nurses performed the bulk of the labor.

**Table 2:** Resources used for D&E and medical induction second-trimester abortion procedures in Western Cape, South Africa.

Category	Resources	
*For all procedure types*:		
Personnel^a^	Professional nurse, staff nurse, ultrasound technician, social worker	
Consumables	Office supplies, hand washing/sanitizing supplies, ultrasound gel, supplies for exam and urine/blood testing, sanitary towels, linen savers, gloves, masks, cotton swabs, etc.	
Medication^b^	Analgesics, antibiotics, anxiolytics, uterotonic medications, misoprostol, etc.	
Equipment	Waiting room furnishings, consulting room furnishings, ultrasound machine, equipment for assessing vital signs, etc.	
Laboratory	Blood sent to national lab, if sent (syphilis, full blood count and Rhesus testing^c^)	
*Additional resources*:	*MI procedures only*	*D&E procedures only*
Personnel^d^	Registrar/intern	Medical officer
Consumables	Supplies for administering medication and managing expulsion	Supplies for paracervical block, etc.
Medication/Devices	Mifepristone^e^	Paracervical block
Equipment	Inpatient hospital furnishings (bed, locker, etc.)	MVA cannulas and aspirator^f^, other small medical equipment, theater/operating room furnishings, recovery room furnishings, etc.

D&E = dilation and evacuation, MVA = manual vacuum aspiration, MI = medical induction

^a^ For international comparability, professional nurses generally have four years of nursing education, and staff nurses have 2 years of education.

^b^ Not all patients received all medication types.

^c^ Rhesus rapid testing (by card) was done at the study D&E site. Lab-based Rhesus testing was used at the medical induction sites only.

^d^ For D&E procedures, the medical officer performed the D&E procedure. For medical induction procedures the registrar conducted the initial assessment, prescribed medication for the medical induction procedure.

^e^ Mifepristone was only required for medical induction performed with mifepristone and misoprostol.

^f^ The aspirator and cannulas were reportedly replaced every 1.2 months.

### Costs and cost-effectiveness

The total average cost per procedure is summarized in Table 3 along with a breakdown of the cost components. D&E was least costly at $88.89 per woman seen, and medical induction with a combined regimen was less costly than induction with misoprostol alone at $298.03 and $364.08 respectively. However, comparing the plausible ranges in costs for the two medical procedures, there was overlap, suggesting that the two procedures may cost the same in some circumstances. Personnel costs were the largest component of the total cost for all procedures.

**Table 3:** Average costs of providing second-trimester safe abortion services in Western Cape, South Africa^a^ (USD 2015).

	Site A: D&E with misoprostol for priming (n = 80)			Site B: Medical induction with mifepristone + misoprostol (n = 178)			Site C: Medical induction with misoprostol only (n = 120)		
	Cost	(Range)^b^	% of total	Cost	(Range)^b^	% of total	Cost	(Range)^b^	% of total
Personnel	51.22	(38.66–63.78)	57.6	238.37	(178.57–298.17)	80.0	321.94	(241.18–402.69)	88.4
*Initial procedure*^c^	*49*.*38*	*(37*.*03–61*.*72)*		*236*.*54*	*(177*.*41–295*.*68)*		*319*.*95*	*(239*.*96–399*.*93)*	
*UPFS personnel*^d^	*1*.*85*	*(1*.*63–2*.*06)*		*1*.*83*	*(1*.*16–2*.*49)*		*1*.*99*	*(1*.*23–2*.*76)*	
Consumables	16.52	(12.39–20.65)	18.6	8.94	(6.70–11.17)	3.0	6.49	(4.87–8.12)	1.8
Medication	2.07	(2.07–2.07)	2.3	21.40	(21.40–21.40)	7.2	4.10	(4.10–4.10)	1.1
Equipment	13.42	(10.06–16.77)	15.1	10.78	(8.08–13.47)	3.6	12.23	(9.17–15.29)	3.4
Laboratory	0.00	(0.00–0.00)	0.0	13.10	(13.10–13.10)	4.4	13.04	(13.04–13.04)	3.6
UPFS facility fee^d^	5.67	(4.25–7.08)	6.4	5.44	(4.08–6.80)	1.8	6.28	(4.71–7.84)	1.7
**Average total cost**	**88.89**	**(67.43–110.35)**	**100.0**	**298.03**	**(231.93–364.12)**	**100.0**	**364.08**	**(277.08–451.09)**	**100.0**

UPFS = Uniform Patient Fee Schedule (South Africa’s published charges for health services in the public sector)

^a^ All incremental costs, including those for extra evacuation procedures required to complete the abortion procedure and complication/hospitalization costs. The denominator is all women. Costs are incremental in that they exclude most overhead costs.

^b^ Ranges in parentheses represent ±25% changes in personnel time, supply, equipment, and hospitalization costs.

^c^ Costs presented for the “initial procedure” include extra evacuation costs if necessary. They exclude costs for managing other complications.

^d^ Costs for hospitalization apply to women with complications only (excluding extra evacuations) and are based on South Africa’s Uniform Patient Fee Schedule, which includes overhead. The UPFS breaks its charges into personnel and facilities.

Table 4 provides the total costs incurred for all women seen at the three sites during the study periods. D&E was most cost effective at $91.17 per complete abortion. Medical induction with the combined regimen was also more cost effective than medical induction with the misoprostol only regimen. The average cost for complicated D&E procedures was higher than the costs for complicated medical induction procedures. However, due to a low complication rate, *uncomplicated* procedures contributed 86.5% of the total D&E costs. At the medical induction sites, where extra evacuations were frequent, a significant proportion of the total costs were attributable to complicated procedures. That said, the actual extra evacuations contributed just 5.8% and 3.3% of the total costs for combined-regimen and misoprostol-only medical inductions respectively.

**Table 4:** Average cost per outcome and total costs (USD 2015) for second-trimester abortion procedures in Western Cape, South Africa.

	D&E (n = 80)	MI combined regimen (n = 178)	MI misoprostol only (n = 120)
Total cost during study	7,111	53,049	43,690
Of total, cost of extra evacuations^a^	49.20 (0.7%)	3,075 (5.8%)	1,451 (3.3%)
Average cost per…			
Complete abortion–all	91.17	298.03	375.31
Uncomplicated	81.99	272.94	348.15
Complicated, extra evac. only	81.99^b^	297.94	372.75
Other complicated	683.05	427.42	499.12
Incomplete abortion^c^	57.39	--	135.53
Proportion of total costs attributable to uncomplicated procedures	86.5%	22.6%	40.0%

D&E = dilation and evacuation, MI = medical induction, evac. = evacuation, w/ = with

^a^ For D&E, two evacuations were performed for women who had expulsion prior to the planned D&E procedure. For medical induction, extra evacuations were those performed after fetal expulsion.

^b^ D&E women who had expulsion and then the D&E cost the same as uncomplicated procedures where D&E was performed without prior expulsion because no extra activities were performed.

^c^ Women who had incomplete abortions at the time of study exit cost less than those with complete abortions because the process was interrupted before the abortion was finished. Women changed their minds, absconded before expulsion, etc.

### Sensitivity analysis

Table 5 provides a listing of the parameters varied for uncertainty and sensitivity analysis and their ranges. The base cost of mifepristone as sold to the public sector in South Africa was $16. Reducing this cost by half reduced the cost of medical induction with mifepristone by just 3% to $290.12 per woman seen. Increasing the rate of complications following D&E to 14% increased the cost per woman seen by 83% to $162.49. Changes to all other parameters listed within the ranges indicated did not change the cost per procedure by more than 25% in either direction. Further, no variation to the parameters changed the ranking of the three procedures in terms of average costs or cost-effectiveness.

**Table 5:** Uncertainty and sensitivity analysis.

Parameter	Base	Range	Source for range^a^	Results: Percent change in costs		
				D&E	MI-combined	MI-miso.
*Uncertainty analysis*						
Personnel time per procedure–all staff^b^	Varies^c^	±25%	--	±13.9%	±20.0%	±22.0%
Professional nurse–D&E	74 minutes	±25%	--			
Professional nurse–MI w/comb. regimen	451 minutes	±25%	--			
Professional nurse–MI w/misoprostol	712 minutes	±25%	--			
Total costs for supplies and equipment	See Table 3	±25%	--	±8.4%	±1.7%	±1.3%
Hospitalization cost for 24 hour stay	$130	±25%	--	±1.8%	±0.7%	±0.6%
*Sensitivity analysis*						
Discount rate	3%	3%, 5%	--	-0.7%	-0.2%	-0.2%
Mifepristone cost (per 200 mg)	$16	$8–16	--	N/A	-2.6%	N/A
MVA aspirator lifespan	37 days	7–37	--	+8.4%	+1.2%	+1.1%
Expulsion rate prior to D&E	3%	0–5%	--	±0.2%	N/A	N/A
Rate of retained products after D&E	0%	0–5%	[28,29]	+7.8%	N/A	N/A
Evacuation following medical induction						
With combined regimen	75.3%	10–75.3%	[28,30]	N/A	-7.9%	N/A
With misoprostol only	56.8%	10–56.8%	[28,29]	N/A	N/A	-4.6%
Complication rate, excludes extra evac.						
D&E	1.3%	0–14%	[28,29,31]	-9.6 to 92.3%	N/A	N/A
Medical induction w/combined regimen	5.1%	0–7%	[28,30]	N/A	-13.0 to 4.9%	N/A
Medical induction w/misoprostol	6.0%	0–7%	[28,29]	N/A	N/A	-2.2 to 0.3%
Loss-to-follow-up rate						
D&E	2.5%	0–2.5%	--	+0.9%	N/A	N/A
Medical induction w/combined regimen	0.00%	0–2.5%	--	N/A	-2.0%	N/A
Medical induction w/misoprostol	1.70%	0–2.5%	--	N/A	N/A	+1.5%

D&E = dilation and evacuation, MI = medical induction, miso. = misoprostol, w/ = with, $ = 2015 US dollars, comb. = combined, add. = additional

^a^ If not indicated, the range is assumed.

^b^ Including doctor, social worker, ultrasound technician, etc. More time was required from the professional nurse than any other cadre of health worker.

^c^ See S1 Workbook, Dashboard

## Discussion

In this analysis, D&E services were the least costly and the most cost-effective per complete abortion. Medical induction with a combined regimen of mifepristone and misoprostol was also less costly and more cost-effective than medical induction performed with misoprostol alone. However, an overlap in the uncertainty cost ranges for the two medical induction procedures suggested that the two may have equivalent costs in some circumstances. Nonetheless, even if the two procedures have equivalent costs, the greater efficacy and shorter inpatient stay with the combined regimen are significant advantages.

It is important to note that we defined the cost-effectiveness outcome, a complete abortion, as women who left the study facility having had a complete abortion–rather than based on the “completeness” of the initial procedure. Our costs also reflect all activities required to complete the procedure in each group. This is to reflect how second-trimester abortion services are often offered in practice. Unless the woman changes her mind, the aim of facility staff is to ensure that the woman leaves the facility when her pregnancy has been safely terminated.

There is little published literature on the costs or cost effectiveness of safe, second-trimester abortion services globally. To our knowledge, there is no such literature documenting costs determined through primary data collection in low- or middle-income countries. This is unfortunate given the power of economic data to serve as leverage for policy changes. Cowett et al (2006) evaluated the cost effectiveness of D&E versus medical induction with misoprostol alone as performed in the United States (US) using quality-adjusted life years (QALYs) as the outcome. The authors found D&E to be less costly and more cost-effective in terms of the cost per QALY [32]. Turok et al (2008) compared US-based facility charges for second-trimester abortion procedures. Medical induction was the most costly procedure at $5,029 (US 2008) as compared to D&E in a low volume setting ($4,625) and D&E in a high volume setting ($1,105) [33]. The authors noted that the difference in D&E costs was largely due to the higher rate of complications in the low volume setting where the physicians had fewer opportunities to perform D&Es and maintain their skills.

This analysis has limitations. The data represent three hospitals in one province and may not be representative of all hospital-based second-trimester abortion in South Africa. We were not able to conduct micro-costing at the misoprostol only site, relying instead on detailed service provision information gathered for a separate study [34] and discussions with staff at the facility. We were also not able to conduct micro-costing for complications due to the rarity of complications during the study. As an alternative, we chose to use published charges for procedures, including hospitalization, in the public sector. Based on prior experience, these published charges may vary somewhat from the “true” costs, so we have included them in our sensitivity analysis. Finally, we did not include overhead costs. Budgeting in the public sector in South Africa is often done separately for infrastructure and service delivery. The costs presented here should be viewed as incremental (i.e. service delivery) costs only.

South Africa’s progressive abortion law obligates the state to make services accessible. Currently in South Africa, despite significant problems with second-trimester access [35], second-trimester services comprise a larger proportion of all abortion services than is observed in other settings. However, even if factors contributing to the high second-trimester rate are addressed, there will always be a need for second-trimester abortion services as women may recognize pregnancy late and often maternal health risks and fetal anomalies are only identified later in pregnancy [36].

It has been argued that D&E is the preferred approach for second-trimester procedures from a clinical perspective [28,37]. Many women also prefer this method [38–40]. However, D&E services are not widely available in South Africa, mostly due to resistance among doctors [2] but also as a result of other structural barriers including poor institutional support for interested providers. This is despite prior research from South Africa showing that D&E services generally have greater capacity to meet demand for abortion services, and thus facilitate better access to services, due to shorter procedure times [7].

Prior comparisons of medical abortion regimens using a combined regimen versus misoprostol alone have shown that differences in costs varied widely depending on the location (and thus prices), the visit schedule and whether indirect costs were included [41]. This analysis indicates that given South Africa’s current reliance on medical induction and barriers to D&E scale-up, addition of mifepristone to the medical induction regimen nationwide in the short to medium term could result in cost savings and greater access to second-trimester abortion services due to shorter inpatient stays. Improved access might also mean earlier access for some women. In the longer term, scale-up of D&E services would provide the greatest efficiency gains in terms of spending on service provision; scale-up could also further improve access to second-trimester services and simultaneously address women’s preferences.

## Supporting information

S1 Workbook Snapshot of analytical model. Model table of contents, decision tree, and dashboard where sensitivity analysis and final outcome calculations were done.
(PDF)
