# Supplementary material for: The costs and cost effectiveness of providing second-trimester medical and surgical safe abortion services in Western Cape Province, South Africa
Source: PLoS One. 2018 Jun 28;13(6):e0197485. doi: 10.1371/journal.pone.0197485 (PMC6023192; doi:10.1371/journal.pone.0197485)
Supplement: S1 Workbook — Model table of contents, decision tree, and dashboard where sensitivity analysis and final outcome calculations were done. (PDF) [file pone.0197485.s001.pdf]

Table of Contents - Second-trimester abortion analysis

NB: Contents listing is hyperlinked to workbook tabs.

**Functional and analytical worksheets**	***Description***
Dashboard (cost summary and sensitivity analysis)	*Summarized listing of cost outcomes with levers/ranges for adjustments for sensitivity analysis*
Decision tree for cost-effectiveness calculations	*Clinical and service parameters X average costs per activity, including unscheduled visits*
Average cost per activity calculations	*Resource usage x unit costs per resource for abortion procedure activities (does not include unscheduled visits)*
Analysis parameters	*Listing of parameters (analysis year, discount rate, etc) which can be varied if desired*
**Research usage and clinical outcomes data**	***Source***
First trimester	*IMF, World Bank, Statistics South Africa, etc*
Clinical service parameters and outcomes	*Study database, provider interviews*
Service volume statistics, D&E	*Study enrollment records*
Service volume statistics, MI	*Study enrollment records*
Staff time - summary calculations	*Calculated based on detailed accounts (see below)*
Staff time - D&E detail	*Provider interviews*
Staff time - MI-mife, detail	*Provider interviews*
Staff time - MI-miso, detail	*Provider interviews*
Consumables usage	*Study database, provider interviews*
Equipment usage	*Study database, provider interviews*
Medication usage	*Study database, provider interviews*
Labs/diagnostics usage	*Study database, provider interviews*
Family planning	*Provider interviews*
**Appendices: Unit cost and other source information**	***Source***
Inflation, depreciation, exchange rates, etc.	*IMF, World Bank, Statistics South Africa, etc*
Personnel costs (per type and per minute)	*Calculated using DPSA tables and general accounting practice guidelines for South Africa*
Personnel salaries	*Department of Public Service Administration (DPSA), South Africa*
Supply costs	*National tenders and other sources*
Equipment costs	*National tenders and other sources*
Medication costs	*Master Procurement Catalog (national)*
Lab/diagnostic costs	*National Health Laboratory Service*
Hospitalization costs	*Uniform Patient Fee Schedule, South Africa*
Complication and completion rates	*Published literature*
Additional lists for model drop-down menus	
Full citations for all data sources	

Dashboard - 2nd trimester abortion analysis

[Back to TOC]

Uncertainty and sensitivity analysis dashboard

Uncertainty analysis	Measured value	UA Range	Adjustment	
Costs				
Staff time	--	± 25%	0%	Reset
			UA_2Tstaff_time	
Supply costs	--	± 25%	0%	Reset
			UA_2T_supp_cost	
Equipment costs	--	± 25%	0%	Reset
			UA_2T_equip_cost	
Hospitalization cost (staff & hotel)	1869	± 25%	0%	Reset
	130		*UA_2T_hosp_cost*	
Hospitalization length (days)	1.0	.5-4.5	1	Reset
LI procs only. Hosp for D&E perf is 2 days.				

*included hospitalization length in SA not UA

Sensitivity analysis	Measured value	SA Range	Adjusted value	
Cost inputs				
Mifepristone cost (200 mg)	227.51	113.76-227.51	0%	Reset
15.81	*Mife_cost*		*SA_mife_cost*	
15.81072441	*227.51*			
Aspirator lifespan (days)	0	7-37	0%	Reset
○ First trimester	*37*		*SA_MVA_asp*	
◉ Second trimester	*37*			
Depreciation	3%	3%, 5%		See analysis parameters page

Service volume, % per service

Service volume				
Tyger	◉ Study total	○ 2013 full volume		
	Median	*IQR*		
Tyger	11	6-15	0%	Reset
	10.5			
GSH	19	11-26.5	0%	Reset
	19.0			
MI-miso	13	7.5-18	0%	Reset
	13.0			

Clinical outcomes

Expulsion rate (pre-D&E)					
		2.6%	0%-5%	0%	Reset
		2.6%		*SA_DE_exp_rate*	
Completion / evacuation rate					
If D&E done…	D&E	100.0%	95-100%	0.00%	Reset
		100.0%		*SA_complete_DE*	
Evac. rate post LI	MI-mife	75.3%	10-75.3%	0.00%	Reset
		75.3%		*SA_LI_mife_evac*	
Evac. rate post LI	MI-miso	56.8%	10-56.8%	0.00%	Reset
		56.0%		*SA_LI_miso_evac*	
Complication (not incomplete)					
	D&E	1.3%	0-14%	0.00%	Reset
		1.3%		*SA_comp_DE*	
	MI-mife	5.1%	0-7%	0%	Reset
		5.1%		*SA_comp_LI_mife*	
	MI-miso	6.0%	0-7%	0%	Reset
		6.0%		*SA_comp_LI_miso*	

LTFU

LTFU	D&E	2.5%	0-2.5%	0%	Reset
		2.5%		*SA_LTFU_DE*	
LTFU	MI-mife	0.00%	0-2.5%	0%	Reset
		0.0%		*SA_LTFU_MI_mife*	
LTFU	MI-miso	1.70%	0-2.5%	0%	Reset
		1.7%		*SA_LTFU_MI_miso*	

Average costs - South African Rand (ZAR) (top) and US dollars (bottom)

D&E - total average cost (ZAR)

	Base estimate		Uncertainty analysis	
	**Cost**	**% of total**	**Low**	**High**
Personnel	737.04	57.6%	556.32	917.76
Pers. IP	710.49	55.5%	532.87	888.11
Pers. Com/Hosp	26.55	2.1%	23.45	29.65
Supplies	237.66	18.6%	178.25	297.08
Medication	29.74	2.3%	29.74	29.74
Equipment	193.04	15.1%	144.78	241.31
Laboratory	0.00	0.0%	0.00	0.00
"Hotel" cost (1 day)	81.56	6.4%	61.17	101.95
**Total**	**1279.05**	**100.0%**	**1526.58**	**2505.60**
**Total cost**	**102,324**		**122,126**	**200,448**

MI mife - total average cost (ZAR)

	Base estimate		Uncertainty analysis	
	**Cost**	**% of total**	**Low**	**High**
Personnel	3430.06	80.0%	2569.53	4290.58
Pers. IP	3403.76	79.4%	2552.82	4254.70
Pers. Com/Hosp	26.30	0.6%	16.71	35.88
Supplies	128.63	3.0%	96.47	160.78
Medication	307.89	7.2%	307.89	307.89
Equipment	155.09	3.6%	116.32	193.86
Laboratory	188.54	4.4%	188.54	188.54
"Hotel" cost (1 day)	78.27	1.8%	58.70	97.84
**Total**	**4288.47**	**100.0%**	**5906.98**	**9530.07**
**Total cost**	**763,348**		**1,051,442**	**1,696,352**

MI - miso - total average cost (ZAR)

	Base estimate		Uncertainty analysis	
	**Cost**	**% of total**	**Low**	**High**
Personnel	4632.57	88.4%	3470.55	5794.59
Pers. IP	4603.89	87.9%	3452.92	5754.86
Pers. Com/Hosp	28.68	0.5%	17.63	39.73
Supplies	93.44	1.8%	70.08	116.80
Medication	59.03	1.1%	59.03	59.03
Equipment	175.96	3.4%	131.97	219.95
Laboratory	187.70	3.6%	187.70	187.70
"Hotel" cost (1 day)	90.30	1.7%	67.73	112.88
**Total**	**5239.00**	**100.0%**	**7457.61**	**12285.54**
**Total cost**	**626,462**		**894,913**	**1,474,265**

D&E - total average cost (USD)

	Base estimate		Uncertainty analysis	
	**Cost**	**% of total**	**Low**	**High**
Personnel	51.22	57.6%	38.66	63.78
Pers. IP	49.38	55.5%	37.03	61.72
Pers. Com/Hosp	1.85	2.1%	1.63	2.06
Supplies	16.52	18.6%	12.39	20.65
Medication	2.07	2.3%	2.07	2.07
Equipment	13.42	15.1%	10.06	16.77
Laboratory	0.00	0.0%	0.00	0.00
"Hotel" cost (1 day)	5.67	6.4%	4.25	7.08
**Total**	**88.89**	**100.0%**	**67.43**	**110.35**
**Total cost**	**7,110.94**		**8,487.13**	**13,930.06**
Cost w/out SA	*88.89*			
% increase	*0.00%*			
% decrease	*0.00%*			

MI mife - total average cost (USD)

	Base estimate		Uncertainty analysis	
	**Cost**	**% of total**	**Low**	**High**
Personnel	238.37	80.0%	178.57	298.17
Pers. IP	236.54	79.4%	177.41	295.68
Pers. Com/Hosp	1.83	0.6%	1.16	2.49
Supplies	8.94	3.0%	6.70	11.17
Medication	21.40	7.2%	21.40	21.40
Equipment	10.78	3.6%	8.08	13.47
Laboratory	13.10	4.4%	13.10	13.10
"Hotel" cost (1 day)	5.44	1.8%	4.08	6.80
**Total**	**298.03**	**100.0%**	**231.93**	**364.12**
**Total cost**	**53,049**		**73,069.61**	**117,887.40**
Cost w/out SA	*298.03*			
% increase	*0.00%*			
% decrease	*0.00%*			

MI - miso - total average cost (ZAR)

	Base estimate		Uncertainty analysis	
	**Cost**	**% of total**	**Low**	**High**
Personnel	321.94	88.4%	241.18	402.69
Pers. IP	319.95	87.9%	239.96	399.93
Pers. Com/Hosp	1.99	0.5%	1.23	2.76
Supplies	6.49	1.8%	4.87	8.12
Medication	4.10	1.1%	4.10	4.10
Equipment	12.23	3.4%	9.17	15.29
Laboratory	13.04	3.6%	13.04	13.04
"Hotel" cost (1 day)	6.28	1.7%	4.71	7.84
**Total**	**364.08**	**100.0%**	**277.08**	**451.09**
**Total cost**	**43,536**		**62,191.67**	**102,453.49**
Cost w/out SA	*364.08*			
% increase	*0.00%*			
% decrease	*0.00%*			

Incremental cost effectiveness ratio calculations

	Success rate	Cost	Incremental cost	Incremental outcome	ICER
MI-mife	100.0%	298.03	209.14	2.5%	8365.56
D&E	97.5%	88.89	-275.20	0.8%	-33023.48
MI-miso	96.7%	364.08	364.08	96.7%	376.64
Do nothing	0%	0	--	--	

	Success rate	Cost	Incremental cost	Incremental outcome	ICER
MI-mife	100.0%	298.03	209.14	2.5%	8365.56
D&E	97.5%	88.89	88.89	97.5%	91.17
MI-miso			dom.	dom.	dom.
Do nothing	0%	0	--	--	

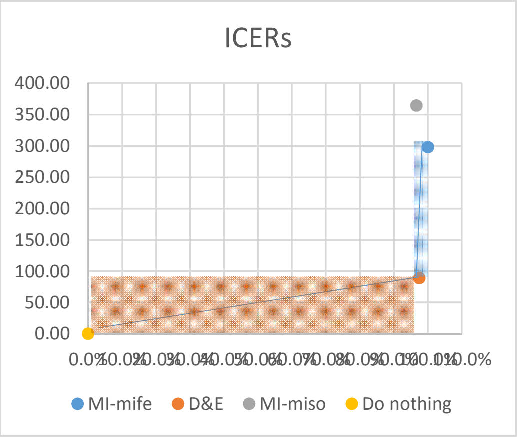

Minutes required per staff type

D&E- staff time

	Base	Low	High
Clerk	0.00	0.00	0.00
Social Worker	17.50	13.13	21.88
Assistant nurse	34.44	25.83	43.06
Staff Nurse	12.38	9.29	15.48
Professional Nurse	74.41	55.81	93.01
PHC Nurse	0.00	0.00	0.00
Nurse midwife	0.00	0.00	0.00
Intern	0.00	0.00	0.00
Registrar	0.00	0.00	0.00
Medical Officer	30.60	22.95	38.25
Specialist	0.00	0.00	0.00
Pharmacist	0.00	0.00	0.00
Ultrasound technician	15.00	11.25	18.75
Cleaner	54.52	40.89	68.15
Security Guard	0.00	0.00	0.00

MI-mife - staff time

	Base	Low	High
Clerk	5.47	4.11	6.84
Social Worker	17.50	13.13	21.88
Assistant nurse	0.00	0.00	0.00
Staff Nurse	451.00	338.25	563.75
Professional Nurse	450.68	338.01	563.36
PHC Nurse	0.00	0.00	0.00
Nurse midwife	0.00	0.00	0.00
Intern	45.00	33.75	56.25
Registrar	73.91	55.43	92.39
Medical Officer	0.00	0.00	0.00
Specialist	0.00	0.00	0.00
Pharmacist	20.00	15.00	25.00
Ultrasound technician	15.00	11.25	18.75
Cleaner	13.68	10.26	17.11
Security Guard	0.00	0.00	0.00

MI-miso - staff time

	Base	Low	High
Clerk	0.00	0.00	0.00
Social Worker	17.50	13.13	21.88
Assistant nurse	0.00	0.00	0.00
Staff Nurse	712.31	534.23	890.38
Professional Nurse	712.00	534.00	890.00
PHC Nurse	0.00	0.00	0.00
Nurse midwife	0.00	0.00	0.00
Intern	45.00	33.75	56.25
Registrar	73.00	54.75	91.25
Medical Officer	0.00	0.00	0.00
Specialist	0.00	0.00	0.00
Pharmacist	10.00	7.50	12.50
Ultrasound technician	15.00	11.25	18.75
Cleaner	20.00	15.00	25.00
Security Guard	0.00	0.00	0.00

Average cost outcomes for costing - 2nd trimester

Back to TOC

D&E

Scheduling - pre 1st visit

	Base estimate		25% higher and lower	
	Cost	% of total	Low	High
Personnel	0	--	0	0
Supplies	0	--	0	0
Medication	0	--		
Equipment	0	--	0	0
Labs/transport	0	--		
**Total**	**0**	**0.0%**	**0**	**0**

First visit

	Base estimate		25% higher and lower	
	Cost	% of total	Low	High
Personnel	372	55.0%	279	464
Supplies	205	30.3%	154	256
Medication	0	0.0%		
Equipment	99	14.6%	74	124
Labs/transport	0	0.0%		
**Total**	**676**	**100.0%**	**507**	**844**

Surgical procedure visit

	Base estimate		25% higher and lower	
	Cost	% of total	Low	High
Personnel	199	56.2%	149	249
Supplies	33	9.4%	25	42
Medication	30	8.6%		
Equipment	91	25.8%	68	114
Labs/transport	0	0.0%		
**Total**	**354**	**100.0%**	**243**	**405**

MI - mife+miso

Scheduling - pre 1st visit

	Base estimate		25% higher and lower	
	Cost	% of total	Low	High
Personnel	21.25	96.7%	16	27
Supplies	0.08	0.3%	0	0
Medication	0	0.0%		
Equipment	1	3.0%	0	1
Labs/transport	0	0.0%		
**Total**	**22**	**100.0%**	**16**	**27**

First visit

	Base estimate		25% higher and lower	
	Cost	% of total	Low	High
Personnel	402	48.0%	302	503
Supplies	15	1.7%	11	18
Medication	228	27.2%		
Equipment	55	6.6%	41	69
Labs/transport	138	16.5%		
**Total**	**838**	**100.0%**	**354**	**590**

Inpatient visit - bottom up costing

	Base estimate		25% higher and lower	
	Cost	% of total	Low	High
Personnel	2761	92.5%	2071	3451
Supplies	89	3.0%	67	111
Medication	57	1.9%		
Equipment	28	1.0%	21	36
Labs/transport	50	1.7%		
**Total**	**2986**	**100.0%**	**2159**	**3598**

MI - miso

Scheduling - pre 1st visit

	Base estimate		25% higher and lower	
	Cost	% of total	Low	High
Personnel	0	--	0	0
Supplies	0	--	0	0
Medication	0	--		
Equipment	0	--	0	0
Labs/transport	0	--		
**Total**	**0**	**0.0%**	**0**	**0**

First visit

	Base estimate		25% higher and lower	
	Cost	% of total	Low	High
Personnel	339	59.2%	255	424
Supplies	15	2.5%	11	18
Medication	0	0.0%		
Equipment	80	14.0%	60	100
Labs/transport	138	24.2%		
**Total**	**573**	**100.0%**	**326**	**543**

Inpatient visit

	Base estimate		25% higher and lower	
	Cost	% of total	Low	High
Personnel	4093	95.4%	3070	5117
Supplies	62	1.4%	46	77
Medication	43	1.0%		
Equipment	43	1.0%	32	53
Labs/transport	50	1.2%		
**Total**	**4291**	**100.0%**	**3148**	**5247**

Administrative management

	Base estimate		25% higher and lower	
	Cost	% of total	Low	High
Personnel	145	96.5%	109	181
Supplies	0	0.1%	0	0
Medication	0	0.0%		
Equipment	5	3.4%	4	6
Labs/transport	0	0.0%		
**Total**	**150**	**100%**	**113**	**188**

Administrative management

Base estimate		25% higher and lower	
Cost	% of total	Low	High
97	97.0%	72	121
0	0.1%	0	0
0	0.0%		
3	2.9%	2	4
0	0.0%		
**100**	**100%**	**75**	**125**

Administrative management

Base estimate		25% higher and lower	
Cost	% of total	Low	High
141	97.1%	106	177
0	0.1%	0	0
0	0.0%		
4	2.9%	3	5
0	0.0%		
**146**	**100%**	**109**	**182**

Hospitalization for PERFORATION includes consultation before admission UPFS

	Base estimate		25% higher and lower	
	Cost	% of total	Low	High
Personnel	2124	24.6%	1593.00	2655.00
Theater/hotel cost	6525	75.4%	4893.75	8156.25
**Total**	**8649**	**100%**	**6486.75**	**10811.25**

Hospitalization for other complication includes consultation before admission, no evac UPFS

	Base estimate		25% higher and lower	
	Cost	% of total	Low	High
Personnel	321	17.2%	240.75	401.25
Hotel cost	1548	82.8%	1161.00	1935.00
**Total**	**1869**	**100%**	**1401.75**	**2336.25**

129.88547

Hospitalization for other complication includes consultation before admission, no evac UPFS

Base estimate		25% higher and lower	
Cost	% of total	Low	High
321	17.2%	240.75	401.25
1548	82.8%	1161.00	1935.00
**1869**	**100%**	**1401.75**	**2336.25**

Hospitalization for other complication includes consultation before admission, no evac UPFS

Base estimate		25% higher and lower	
Cost	% of total	Low	High
321	17.2%	240.75	401.25
1548	82.8%	1161.00	1935.00
**1869**	**100%**	**1401.75**	**2336.25**

Average costs - totals

LTFU after first visit 3% 2/80

Personnel	516	62.5%		
Initial	516	62.5%		
Hospital	0	0.0%		
Supplies	205	24.8%		
Medication	0	0.0%		
Equipment	104	12.6%		
Labs/transport	0	0.0%		
Hospital, other	0	0.0%		
Total (ZAR)	**826**	100.0%		
Total (USD)	**57.39**			

2% 2/120

481	66.9%		
481	66.9%		
0	0.0%		
15	2.0%		
0	0.0%		
84	11.8%		
138	19.3%		
0	0.0%		
**719**	100.0%		
**49.93**			

Expulsion prior to D&E 3% 2/78

Personnel	715	60.6%		
Initial	715	60.6%		
Hospital	0	0.0%		
Supplies	238	20.2%		
Medication	30	2.6%		
Equipment	195	16.6%		
Labs/transport	0	0.0%		
Hospital, other	0	0.0%		
Total (ZAR)	**1180**	100.0%		
Total (USD)	**81.99**			

Scheduling visit costs 20% 35.6/178

21	96.7%		
21	96.7%		
0	0.0%		
0	0.3%		
0	0.0%		
1	3.0%		
0	0.0%		
0	0.0%		
**22**	100.0%		
**1.53**			

LTFU before LI was finished 2% 2/118

481	9.6%		
4574	91.3%		
0	0.0%		
76	1.5%		
43	0.9%		
127	2.5%		
189	3.8%		
0	0.0%		
**5010**	18.3%		
**348.15**			

No complication, no evac 99% 75/76

Personnel	715	60.6%		
Initial	715	60.6%		
Hospital	0	0.0%		
Supplies	238	20.2%		
Medication	30	2.6%		
Equipment	195	16.6%		
Labs/transport	0	0.0%		
Hospital, other	0	0.0%		
Total (ZAR)	**1180**	100.0%		
Total (USD)	**81.99**			

25% 44/178

3260	83.1%		
3260	83.1%		
0	0.0%		
104	2.6%		
285	7.3%		
86	2.2%		
189	4.8%		
0	0.0%		
**3923**	100.0%		
**272.63**			

43% 50/116

4574	91.3%		
4574	91.3%		
0	0.0%		
76	1.5%		
43	0.9%		
127	2.5%		
189	3.8%		
0	0.0%		
**5010**	100.0%		
**348.15**			

Evac + no other complication 0% 0/76

Personnel	1236	36.3%		
Initial	915	26.9%		
Hospital	321	9.4%		
Supplies	272	8.0%		
Medication	61	1.8%		
Equipment	287	8.4%		
Labs/transport	0	0.0%		
Hospital, other	1548	45.5%		
Total (ZAR)	**3403**	100.0%		
Total (USD)	**236.47**			

70% 125/178

3459	80.9%		
3459	80.9%		
0	0.0%		
137	3.2%		
315	7.4%		
178	4.2%		
189	4.4%		
0	0.0%		
**4277**	100.0%		
**297.23**			

51% 59/116

4773	89.0%		
4773	89.0%		
0	0.0%		
110	2.0%		
74	1.4%		
218	4.1%		
189	3.5%		
0	0.0%		
**5364**	100.0%		
**372.75**			

Complication - PERFORATION, no evac 1% 1/76

Personnel	2839	28.9%		
Initial	715	7.3%		
Hospital	2124	21.6%		
Supplies	238	2.4%		
Medication	30	0.3%		
Equipment	195	2.0%		
Labs/transport	0	0.0%		
Hospital, other	6525	66.4%		
Total (ZAR)	**9829**	100.0%		
Total (USD)	**683.05**			

Complication - ANY, no evac 0%

3581	61.8%		
3260	56.3%		
321	5.5%		
104	1.8%		
285	4.9%		
86	1.5%		
189	3.3%		
1548	26.7%		
**5792**	100.0%		
**402.52**			

Complication - ANY, no evac 1% 1/116

4895	71.2%		
4574	66.5%		
321	4.7%		
76	1.1%		
43	0.6%		
127	1.8%		
189	2.7%		
1548	22.5%		
**6879**	100.0%		
**478.04**			

Complication - OTHER, no evac 0% 0/76

Personnel	1036	34.0%		
Initial	715	23.5%		
Hospital	321	10.5%		
Supplies	238	7.8%		
Medication	30	1.0%		
Equipment	195	6.4%		
Labs/transport	0	0.0%		
Hospital, other	1548	50.8%		
Total (ZAR)	**3049**	100.0%		
Total (USD)	**211.87**			

Evac + complication

				0% 0/76				5% 9/178				5% 6/116
Personnel	1236	36.3%			3780	61.5%			5094	70.4%		
Initial	915	26.9%			3260	53.0%			4574	63.2%		
Hospital	321	9.4%			520	8.5%			520	7.2%		
Supplies	272	8.0%			137	2.2%			110	1.5%		
Medication	61	1.8%			315	5.1%			74	1.0%		
Equipment	287	8.4%			178	2.9%			218	3.0%		
Labs/transport	0	0.0%			189	3.1%			189	2.6%		
Hospital, other	1548	45.5%			1548	25.2%			1548	21.4%		
Total (ZAR)	**3403**	100.0%			**6146**	100.0%			**7233**	100.0%		
Total (USD)	**236.47**				**427.12**				**502.64**			

All patients (with weighted average costs based on frequency of incomplete, etc.)

Personnel	737	57.6%			3430	80.0%			4633	88.4%		
Initial	710	55.5%			3404	79.4%			4604	87.9%		
Hospital	27	2.1%			26	0.6%			29	0.5%		
Supplies	238	18.6%			129	3.0%			93	1.8%		
Medication	30	2.3%			308	7.2%			59	1.1%		
Equipment	193	15.1%			155	3.6%			176	3.4%		
Labs/transport	0	0.0%			189	4.4%			188	3.6%		
Hospital, other	82	6.4%			78	1.8%			90	1.7%		
Total (ZAR)	**1279**	100.0%			**4288**	100.0%			**5239**	100.0%		
Total (USD)	**88.89**				**298.03**				**364.08**			

Decision tree - 2nd trimester abortion analysis

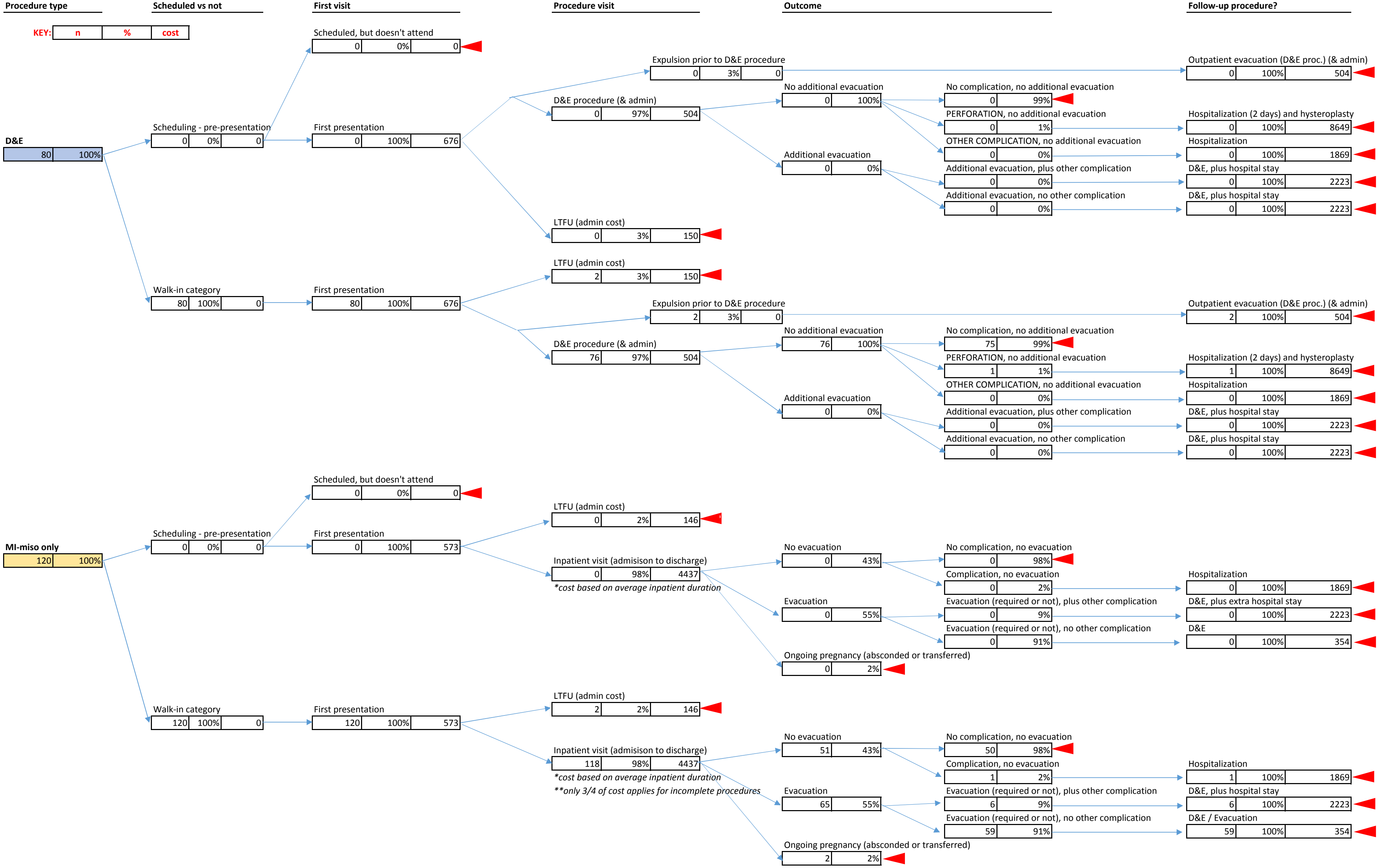

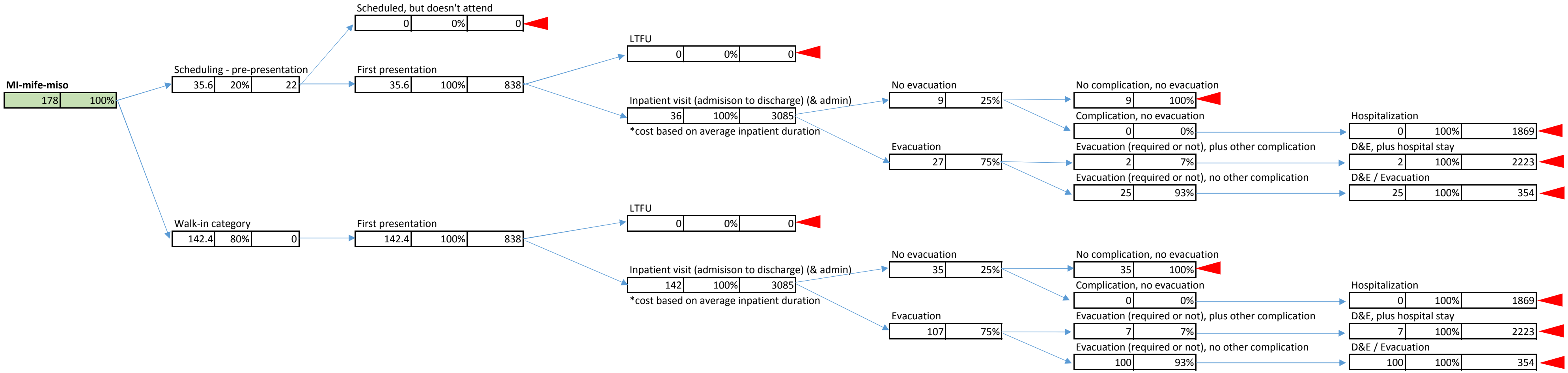
MI-mife-miso
178 100%
Scheduling - pre-presentation
35.6 20% 22
Scheduled, but doesn't attend
0 0% 0
First presentation
35.6 100% 838
LTFU
0 0% 0
Inpatient visit (admisison to discharge) (& admin)
36 100% 3085
*cost based on average inpatient duration
No evacuation
9 25%
Evacuation
27 75%
No complication, no evacuation
9 100%
Complication, no evacuation
0 0%
Evacuation (required or not), plus other complication
2 7%
Evacuation (required or not), no other complication
25 93%
Hospitalization
0 100% 1869
D&E, plus hospital stay
2 100% 2223
D&E / Evacuation
25 100% 354
Walk-in category
142.4 80% 0
First presentation
142.4 100% 838
LTFU
0 0% 0
Inpatient visit (admisison to discharge) (& admin)
142 100% 3085
*cost based on average inpatient duration
No evacuation
35 25%
Evacuation
107 75%
No complication, no evacuation
35 100%
Complication, no evacuation
0 0%
Evacuation (required or not), plus other complication
7 7%
Evacuation (required or not), no other complication
100 93%
Hospitalization
0 100% 1869
D&E, plus hospital stay
7 100% 2223
D&E / Evacuation
100 100% 354

Costs

ZAR	USD
0.00	0.00
0.00	0.00
0.00	0.00
0.00	0.00
0.00	0.00
0.00	0.00
0.00	0.00
0.00	0.00
1651.61	114.78
2359.56	163.98
88483.64	6149.14
9828.78	683.05
0.00	0.00
0.00	0.00
0.00	0.00

D&E

Total cost ZAR 102,323.60 | **Total cost USD** 7,110.94

Total average cost per woman seen
1,279.05 | 88.89

Complete cases (with or without complication)
78

Cost per complete case (with or without complication)
1,311.84 | 91.17

Complicated cases (excluding re-evac's and expulsion)
1

Average cost per complicated case
9,828.78 | 683.05

LTFU/ongoing pregnancy
2

Average cost 825.81 | Average cost 57.39

Complete, uncomplicated cases (excludes re-evac and expulsion)
75

Average cost per complete, uncomplicated case
1,179.78 | 81.99

	% of total	inverse
6,149.14	86.5%	13.5%

Cases with expulsion
2

Average cost per case with prior expulsion
1179.78 | 81.99

Added cost for extra D&E:
24.60 ea. | *49.20 total* | 0.7%

ZAR	USD
0.00	0.00
0.00	0.00
0.00	0.00
0.00	0.00
0.00	0.00
0.00	0.00
0.00	0.00
1437.02	99.86
250488.01	17407.57
6878.76	478.04
43396.41	3015.82
316460.38	21992.30
7801.12	542.14

MI-miso only

Total cost ZAR 626,461.70 | **Total cost USD** 43,535.73

Total average cost per woman seen
5,220.51 | 362.80

Complete cases (with or without complication)
116

Cost per complete case (with or without complication)
5,400.53 | 375.31

Complicated cases (excludes ongoing preg's, excludes evac without complication)
7

Average cost per complicated case
7,182.17 | 499.12

Complete, uncomplicated cases (excludes re-evac)
50

Average cost per complete, uncomplicated case
5,009.76 | 348.15

	% of total	inverse
17,407.57	40.0%	60.0%

Cases with evac (no complication)
59

Average cost per case with prior expulsion
5363.74 | 372.75

Added cost for extra D&E:
24.60 ea. | *1,451.36 total* | 3.3%

Ongoing pregnancy
4

Average cost 2309.53 | Average cost 135.53

0.00	0.00
0.00	0.00
34718.07	2412.72
0.00	0.00
11102.39	771.56
107475.40	7468.96
0.00	0.00
138098.19	9597.08
0.00	0.00
44251.22	3075.22
427702.54	29723.03

MI-mife-miso

**Total cost ZAR**	**Total cost USD**
763,347.81	53,048.58

Total average cost per woman seen

4,288.47	298.03

Complete cases (with or without complication)

178

Cost per complete case (with or without complication)

4,288.47	298.03

Complicated cases (includes ongoing preg's, excludes evac without complication)

9

Average cost per complicated case

6,150.47	427.42

Complete, uncomplicated cases (excludes re-evac)

44

Average cost per complete, uncomplicated case

			% of total	inverse
3,927.50	272.94	12,009.80	22.6%	77.4%

Cases with evac (no complication)

125

Average cost per case with prior expulsion

4281.47	297.54	*Added cost for extra D&E:*

24.60	3,074.88	5.8%
ea.	*total*	
